# COVID-19 – Changes in Workload and Clinical Practice in Trauma and Orthopaedics in a District General Hospital in the United Kingdom

**DOI:** 10.5704/MOJ.2103.015

**Published:** 2021-03

**Authors:** G Faria, S Virani, BJ Tadros, BS Dhinsa, G Reddy, J Relwani

**Affiliations:** Department of Orthopaedics, East Kent Hospitals University NHS Foundation Trust, Ashford, United Kingdom

**Keywords:** COVID-19, response, trauma

## Abstract

**Introduction:**

COVID-19 has had a significant impact on the entire health system. The trauma and orthopaedic service has been compelled to alter working practices to respond proactively and definitively to the crisis. The aim of this study is to summarise the impact of this outbreak on the trauma and orthopaedic workload and outline the response of the department.

**Materials and Methods:**

We retrospectively collected data comparing patient numbers pre-COVID-19, and prospectively during the early COVID-19 pandemic. We have collected the numbers and nature of outpatient orthopaedic attendances to fracture clinics and elective services, inpatient admissions and the number of fracture neck of femur operations performed.

**Results:**

The number of outpatient attendances for a musculoskeletal complaint to Accident and Emergency and the number of virtual fracture clinic reviews reduced by almost 50% during COVID-19. The number of face-to-face fracture clinic follow-ups decreased by around 67%, with a five-fold increase in telephone consultations. Inpatient admissions decreased by 33%, but the average number of fracture neck of femur operations performed has increased by 20% during COVID-19 compared to pre-COVID-19 levels.

**Conclusion:**

We have noted a decrease in some aspects of the trauma and orthopaedic outpatient workload, such as leisure and occupational-related injuries but an increase in others, such as fracture neck of femurs. Many injuries have significantly reduced in numbers and we consider that a model could be developed for treating these injuries away from the acute hospital site entirely, thereby allowing the acute team to focus more appropriate major trauma injuries.

## Introduction

COVID-19 has had a significant impact on the health services since late December 2019, when the outbreak was reported in Wuhan, China^1^. By mid-March, the United Kingdom government had introduced several public measures to try and restrict the spread of COVID-19^2^. This was primarily based on social distancing and self-isolation.

Our Trust includes two acute hospital sites with trauma capabilities and four peripheral minor injuries units. All sites have had to alter their working practice to deal with the escalating crisis. The orthopaedic department also had to assist front-line specialties (such as intensive care) within the hospital deal with the anticipated large number of COVID-19 positive patients needing admission. Alterations have been made based on the British Orthopaedic Association (BOA) Standards for Trauma (BOAST) COVID-19 Guidance^3^ and local consultant consensus.

These changes included having new outpatient and fracture clinics that were conducted by telephone or video consults and would only meet patients face-to-face when necessary (e.g. plaster change, wound reviews). Where possible, patients had their radiographs on an outpatient basis in local “COVID-19-clean” sites and contacted the next day. To reduce the need for attendance to hospital, patients were placed into a splint/boot in preference to plaster if the fracture pattern and patient demographics allowed for it.

For inpatient services, elective lists were cancelled within a week of the measures being implemented, but trauma and emergency service provision continued uninterrupted. The trauma lists were split into ambulatory and non-ambulatory trauma. The non-ambulatory trauma ran on the main sites with appropriate adaptations discussed below, whilst ambulatory trauma patients were sent home after being assessed and allocated to a separate list in a “COVID-19-clean” site if a negative COVID-19 swab was produced.

Surgical practice modifications were made. Staff safety measures implemented included the reduction of use of saws, drills, diathermy and pulsatile lavage as these were identified as aerosol-generating procedures^4^. Despite a recent research report published in Nature showing that there is no viral load detected in blood of infected cases^5^, our Trust has recommended that all staff performing any orthopaedic procedure should use full personal protective equipment (PPE), including filtering facepiece 3 (FFP3) masks, and treat every patient as COVID-19 positive. Also, the number of staff members in theatre was reduced to the absolute minimum necessary to perform the operation.

Efficiency and Capacity Measures were implemented in line with Public Health England guidance^2^. These included certain theatre practices such as the ‘donning and doffing’ of PPE, recovering patients in the same theatre, and once the patient had left the theatre complex, deep cleaning performed after every case. By the second week of these measures being implemented a second theatre was provided to alternate patients between them to improve efficiency without the need for additional personnel.

The aim of this study is to summarise the impact of this outbreak on the trauma and orthopaedic workload and document the immediate response and change in work patterns of the department to provide colleagues with measures we have implemented to prepare for a potential second wave of COVID-19.

## Material and Method

We collected data to compare patient numbers in the immediate period pre-COVID (retrospective) and during the pandemic (prospective). Data was collected and averaged from 16th March 2019 to 14th April 2019 (‘normal’ practice pre-COVID-19) and data from 16th March 2020 to 14th April 2020 (during COVID-19 measures).

Data was collected from Accident and Emergency documentation software (ECasCard), AllScripts PAS patient system and Theatreman [Theatre Management System].

Outpatient data collected included: total number of musculoskeletal attendances to Accident and Emergency, attendances to Accident and Emergency for which an orthopaedic referral was sought, nature of referrals from Accident and Emergency to orthopaedics, numbers of patients reviewed in the Virtual Fracture Clinic (VFC) and numbers of patients who were consulted via telephone versus face-to-face consultations.

Inpatient data collected included: number of inpatient admissions and average number of fracture neck of femur operations performed in the month of April 2019 (pre-COVID-19) compared to April 2020 (during COVID-19).

STROBE Guidelines were consulted and the manuscript was prepared in accordance with the checklist for observational studies. All data was tabulated in an Excel sheet [Microsoft Corp, Redmond, WA]. This was followed by calculation of the mean, standard deviations and standard errors of the mean. Paired t-test was applied to determine statistical significance assuming 95% confidence intervals.

## Results

**Fig. 1:**
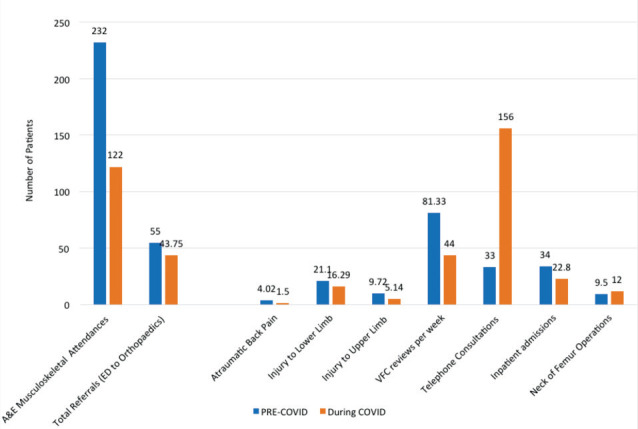
Graph showing the change in numbers of patients being referred to Trauma and Orthopaedics, neck of femur fractures and number of virtual fracture clinic and telephone consultations (per week).

The change in the workload of the department is summarised in (Fig. 1). Of special note is the reduction in number of Accident and Emergency (A&E) attendances with a musculoskeletal complaints, lower limb referrals and inpatient admissions but an increase in neck of femur fractures presenting to the department. A significant increase in telephone consultations from 33 (+/-8.98) to 156 (+/-27.4) is also noticeable (p=0.0015).

**Fig. 2:**
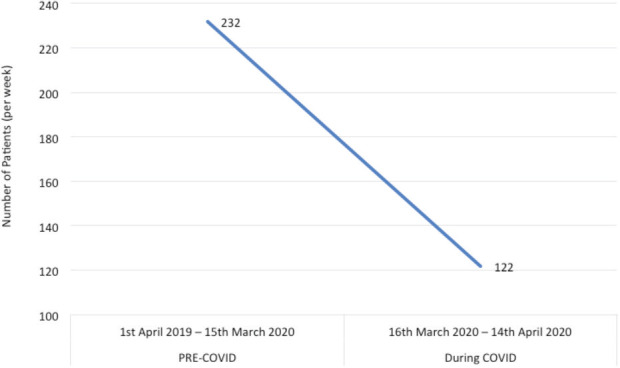
Graph showing decline in total A&E attendances with a musculoskeletal complaint.

**Fig. 3:**
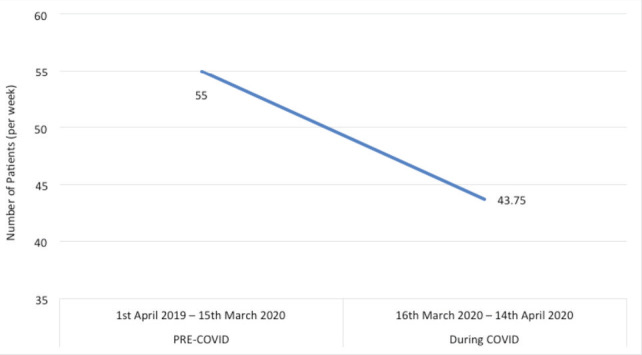
Average referrals from Accident and Emergency to trauma and orthopaedics (per week).

The total number of A&E attendances for a musculoskeletal complaint were average 232 per week pre-COVID-19 and 122 per week during COVID-19 (p<0.05) (Fig. 2). The average number of referrals from Accident and Emergency to trauma and orthopaedics decreased from 55 per week prior to the COVID-19 pandemic to 43.75 per week during the COVID-19 pandemic to date (p<0.05). This includes both inpatient and outpatient referrals (Fig. 3).

The nature of referrals from A&E to Trauma and Orthopaedics: Referrals for atraumatic back pain decreased from an average of 4.02 per week (pre-COVID-19) to 1.5 per week (during the COVID-19 pandemic) (p<0.05). Average referrals in the injury to lower limb category reduced from 21.1 per week pre-COVID-19 to 16.29 per week during COVID-19 (p<0.05), including fracture neck of femur. Referrals for injury to upper limb reduced from average 9.72 per week to average 5.14 per week (p<0.05). A summary of these results is presented in (Fig. 1).

The average number of virtual fracture clinic (VFC) reviews declined by almost half from 81.33 on average per week pre-COVID-19 to on average 44 per week during COVID-19 measures and this difference was statistically significant (p<0.05). This data is presented in summary in (Fig. 1).

**Fig. 4:**
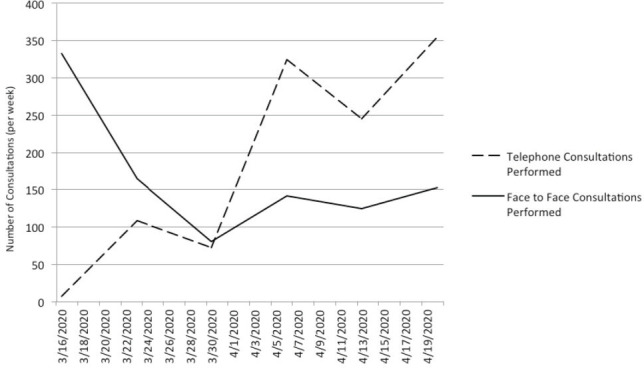
Graph showing the increase in number of telephone consultations across fracture and elective clinics and decrease in the number of face-to-face consultations throughout COVID measures (per week).

The number of telephone consultations performed increased exponentially from average 33 per week pre-COVID-19 (which comprised Surgical Care Practitioner follow up clinics only) to average 155 per week during COVID-19, with numbers reaching over 350 as the uptake increased. There is also reciprocal decline in the number of face-to-face reviews (Fig. 4). These are a mixture of new and follow up patients.

Inpatient admissions significantly declined from 34 on average per week pre-COVID-19 to 22.8 per week (p<0.05) (Fig. 1). These include patients with fracture neck of femur and other conditions which required inpatient treatment such as intravenous antibiotics or operative management, or non-ambulatory patients.

The average number of fracture neck of femur operations (including dynamic hip screw, hemiarthroplasty, total hip arthroplasty and intramedullary nail fixation) increased from average 38 per month performed during April 2019 (pre-COVID-19) to 47 per month during April 2020 (during COVID-19) (Fig. 1). This is despite a decline in the number of lower limb referrals from A&E and a reduction in inpatient admissions.

## Discussion

The data from our hospital reveals interesting results, as summarised in (Fig. 1). The number of patients presenting to the Accident and Emergency department as well as the patients referred to the Trauma and Orthopaedics department have reduced. Subsequently, the number of orthopaedic inpatients has also diminished. Among the orthopaedic referrals we have noted a decline in all types of referrals including atraumatic back pain, as well as upper and lower limb injuries. These injuries were analysed as they make up the majority of our on-call referral load. It is interesting to note that the decline in back pain referrals has been most precipitous with average number of referrals reducing to less than half of previous numbers. Despite the fall in lower limb injuries we have noted an approximately 30% rise in the neck of femur fractures when compared to a similar period last year. Neck of femur fractures are the only skeletal injuries that have seen an uptrend during this period. A possible cause for this could be a reduction in physical support usually provided by relatives and carers for these elderly and frail patients, leading to increased prevalence of trauma and hip fractures^6^. The national lockdown and introduction of ‘shielding’ to protect the clinically vulnerable could account for this.

There are a number of potential reasons for the above-mentioned trends. Firstly, there has been a reduction in leisure and outdoor activities as mandated by government regulations^2^ and therefore a reduced likelihood for patients to injure themselves, although no evidence is available in the literature regarding this. Moreover, occupational injuries and physical exertion related spinal problems would be expected to have reduced.

We also believe that there could be a component of hesitancy and apprehension from patients to seek medical attention due to fear of contracting COVID-19 if they present to the hospital. Again, there is no evidence available for this, but should this be the case, one would expect a surge in these cases presenting to hospital once the lockdown is eased, and this requires appropriate preparation to tackle the increased demand. One also wonders if these patients might, under normal circumstances, present to the A&E more frequently than is ‘essential’ as it may be the most convenient and quickest way to access care, albeit for a chronic problem (which should not be the remit of accident and emergency departments).

Significant changes were made to the running of clinics. These include all clinics being conducted by telephone consultation and only seeing patients face-to-face when absolutely necessary and appropriate assessment could not take place over the phone (e.g. plaster change, wound reviews). Where possible patients had their radiographs on an outpatient basis in the local community hospital (COVID-19-negative) and patients contacted with the results. Patients attending for change/removal of plaster were either discharged directly or reviewed face-to-face depending on clinical need. To reduce the need of attending hospital, patients with certain minimally-displaced or stable fractures (such as extra-articular distal radius) were placed into a splint/boot in preference to plaster if possible. Some clinicians are using a video consultation system, “AccuRX” [AccuRx, London, U.K] for patient review. This is a useful tool allowing limited clinical examination and wound reviews.

For the patients that were required to attend hospital, strict infection control policies were introduced. For example, patients were required to wear face masks and sanitise hands on entry to the hospital and had their temperature checked. In order to reduce the number of people in the building at a time, patients were asked to attend on their own and wait outside the hospital until their appointment time. On arrival to the fracture clinic, a list of pre-determined questions was asked to ascertain risk of COVID-19 (such as presence of fever or loss of taste or smell). In addition, staff members were required to abide by strict protocols including use of PPE for each patient (mask, gloves and apron) and regular hand-washing.

A study to assess the effectiveness of using telemedicine would be useful going forward. Feedback from patients has been encouraging so far and it seems likely that telemedicine could become an integral part of our future clinical practice^7,8^. Clinics conducted by telephone or video conferencing allow accurate and timely consultations which often result in a management plan going forward^9^. Patients appear to be receptive to the idea of communicating with their clinicians via technology, especially in light of the risk of attending hospital in the current pandemic^9^. However, some problems were encountered, such as failure of timing of consultation in conjunction with relevant imaging, difficulty discerning certain pathologies due to the inability to examine patients and difficulty with determining patients’ suitability for surgery over the telephone.

Elective clinics are still carried out, using the same principles and if appropriate, patients are being placed directly onto the waiting list from the telephone clinic. However, all such patients listed for surgery are only done so after discussion with the operating consultant. They will be reviewed by the operating surgeon at the pre-assessment clinic.

We have seen that inpatient admissions to Trauma and Orthopaedics wards also declined during COVID-19 to date. For inpatient trauma, we attempted to expedite the time to operation to reduce the length of hospital stay and associated morbidity and mortality of these patients. However, we encountered difficulties such as reduced trauma list operating time, increased patient turnaround times^2^ and a reduced number of theatre staff (as a result of redeployment to other areas such as intensive care). Due to the decreased orthopaedic workload in our hospital, a number of junior orthopaedic staff were asked to assist in the intensive care unit in order to deal with the rising number of cases. In other institutions, orthopaedic staff were also redeployed to Accident and Emergency and General Medicine^10^, as well as intensive care. There was significant concern amongst the staff about prolonged exposure to COVID-19 in this situation and many members of staff tested positive for COVID-19 during their redeployment to these high-risk areas^10^.

A study to ascertain the effect of these changes is currently ongoing, subject to data availability once the COVID-19 pandemic has passed.

## Conclusion

Life as we know it as changed with the COVID-19 pandemic. All healthcare services across the globe have had to rapidly adapt and alter their services to this evolving situation. Some clinical tools explored in these times could become an integral part of our future practice. We believe analysis of further data of clinical workload and quantitative evaluation of efficacy of various new clinical tools needs to be pursued to build an international consensus and robust framework to cater to clinical needs of the population.
